# Ferroptosis-Associated Classifier and Indicator for Prognostic Prediction in Cutaneous Melanoma

**DOI:** 10.1155/2021/3658196

**Published:** 2021-10-28

**Authors:** Hao Zeng, Cong You, Leran Zhao, Jiangyi Wang, Xiaoying Ye, Tao Yang, Chunlei Wan, Longying Deng

**Affiliations:** ^1^Department of Burn and Plastic Surgery, Ganzhou People's Hospital, The Affiliated Ganzhou Hospital of Nanchang University, Ganzhou, China; ^2^Department of Dermatology and Venereology, Candidate Branch of National Clinical Research Centre for Skin and Immune Diseases, The First Affiliated Hospital of Gannan Medical University, Ganzhou, China; ^3^Department of Dermatology and Venereology, The General Hospital of Tianjin Medical University, Tianjin, China; ^4^Department of Gastrointestinal Surgery, The First Affiliated Hospital of Gannan Medical University, Ganzhou, China

## Abstract

Ferroptosis plays a critical role in different types of cancers, but the prognostic impact of ferroptosis in cutaneous melanoma remains lacking. Therefore, ferroptosis-related genes (FRGs) were firstly obtained from the FerrDb database and the differentially expressed FRGs were identified by the “limma” algorithm. Next, the prognostic differentially expressed FRGs were screened out by univariate Cox regression, which were subsequently used to cluster melanomas into two subtypes (clusters A and B). Besides, the Boruta algorithm and principal component analysis (PCA) were performed to build a 15-FRGs indicator, which can robustly predict patients' overall survival (OS) and be considered as an independent prognostic factor in melanoma. The melanoma patients were further divided into high- and low-FRGs score groups. The high score group have a good prognosis, with higher T cell immune infiltrating and lower mutation frequencies in NRAS, KRAS, and NF1. Finally, we discovered that many immune processes and several chemotherapy drugs were closely associated with FRGs score. Thus, our study provides a novel ferroptosis-associated classifier and indicator to predict the prognosis of melanoma. Besides, we identified several potential chemotherapy drugs to induce ferroptosis and could supply additional effective treatments.

## 1. Introduction

Melanoma is a highly lethal cutaneous tumor which originates from the malignant transformation of melanocytes. Although melanoma takes 5% incidence in all skin-related cancer patients, it causes an overall mortality rate of 80%. Due to the absence of early symptoms, melanomas are frequently diagnosed at an advanced stage and only 10% of patients have 5-year survival [1, 2]. Currently, the traditional system for melanoma treatment and prognostic prediction, such as Clark level, tumor stage, and histological type, is growingly becoming difficult to illustrate the diversity of clinical outcomes [3]. Molecular features such as gene transcription, translation, and many posttranslational modifications lead to heterogeneity of cutaneous melanoma [4]. Thus, it is urgently required to explore the new biomarkers for classification and early predicting the prognosis of melanoma.

Ferroptosis is defined as a new type of programmed cell death based on iron, which is characterized by the accumulation of lethal lipid peroxides and intracellular reactive oxygen species (ROS) production [5]. In recent years, many significant studies have demonstrated that ferroptosis is a participant in a large number of pathological processes, especially in the proliferation and growth of cancer cells [6, 7]. The activation of ferroptosis suppressed the development of many chemotherapy-resistant cancers, which indicated that ferroptosis may be a promising therapeutic target for cancer treatment [8]. Except for induction agents of ferroptosis, various genes have also been identified as drivers or suppressors of ferroptosis [9]. For example, the downregulation of SLC7A11 and SLC3A2 induces the activation of ferroptosis and leads to antitumor efficacy [10]. Besides, the prognostic value of ferroptosis-related genes has been demonstrated in much cancer research. For instance, Liu et al. developed a 19-ferroptosis-related gene signature to predict the survival of glioma patients [11]. Lou and Ma constructed a novel 7 ferroptosis-associated prognostic genes' indicator for uveal melanoma [12]. Zhang et al. identified 15 ferroptosis-related mRNAs therapeutic targets for the treatment of ovarian cancer [13]. Similarly, ferroptosis-associated studies are increasing to reveal its significance in the progression of cutaneous melanoma [14]. Zhang et al. proved that the silence of miR-9 promotes ferroptosis in melanoma cells. Basit et al. reported that mitochondrial complex I inhibitor is an important agent to induce ferroptosis in melanoma cells [15]. However, a comprehensive analysis of the ferroptosis-related gene in the prognosis of cutaneous melanoma patients remains lacking. Fortunately, the availability of public, large-scale datasets such as The Cancer Genome Atlas (TCGA) and Gene Expression Omnibus (GEO) databases provided numerous transcriptome profiles to investigate the landscape of ferroptosis-related genes.

Therefore, in this research, we comprehensively evaluated the features of ferroptosis-related genes in cutaneous melanoma according to the TCGA and GEO RNA-Seq datasets. Based on the expression level of ferroptosis-related genes, melanoma patients were successfully classified into tumor subtypes with different clinical characteristics and survival events. Next, we constructed a prognostic signature with 15 ferroptosis-related genes, which well predicted the survival outcome of melanoma patients. Finally, several chemotherapy drugs were identified from the CellMiner database [16], which intimately associated ferroptosis signature and afforded alternative therapies for system treatment of melanoma.

## 2. Materials and Methods

### 2.1. Datasets Acquisition and Analysis

The RNA-Seq datasets and clinical features referred to in this study were acquired from the publicly available databases. The normal skin tissue-matched melanoma tumor dataset (TCGA-GTEx) was downloaded from the Xena website (https://xena.ucsc.edu/public-hubs/). The RNA expression datasets that contained GSE15605, GSE3189, and GSE46517 were derived from the GEO database (https://www.ncbi.nlm.nih.gov/geo). Then, the data form of gene expression for these datasets was converted to TPMs (transcripts per kilobase million). Next, “ComBat” algorithm was performed to reduce the batch effect and merge these datasets into a large cohort. Besides, another two melanoma datasets (GSE19345 and GSE65904) with survival information were selected out for outside validation analyses.

### 2.2. Ferroptosis-Related Genes

In total, 177 ferroptosis-related genes (FRGs) consisting of 108 driver genes and 69 suppressor genes were downloaded from the FerrDb website (https://www.zhounan.org/ferrdb) which deposited regulators and markers of ferroptosis collected from the PubMed database [17]. After removing the overlapped genes, 173 FRGs were finally selected out for further analysis.

### 2.3. Differently Expressed FRGs

Samples in the large cohort were classified into normal and tumor groups. “Limma” R package was used to screen the differently expressed FRGs with adjusted *P* < 0.05 and |log 2 fold change (FC)| ≥0.5. Next, Gene Ontology (GO) enrichment analyses were respectively performed to explore the underlying molecular mechanism in upregulated and downregulated FRGs.

### 2.4. FRGs-Associated Subtype Identification

Univariate Cox regression analysis was used to screen the prognostic value of differently expressed FRGs in the TCGA-SKCM dataset, where *P* < 0.05 was regarded as statistically significant. Afterward, we utilized the consensus clustering method to generate the final clustering of patients based on the prognostic FRGs. Principal component analysis (PCA) was performed to distinguish patient clusters distribution in the first two principal components. Moreover, we also explored interactive correlation among these prognostic FRGs. To test the stability of FRGs for classification, patients in GSE19345 and GSE65904 datasets were accordingly classified and verified.

### 2.5. Feature Selection and FRGs Score Construction

Furthermore, the Boruta algorithm was performed to select the important features from the identified prognostic FRGs. These important FRGs were further recruited to PCA calculation and the principal component 1 (PC1) was extracted to represent the signature score. Finally, we constructed FRGs associated prognostic model in TCGA-SKCM datasets and the formula is listed as follows: FRGs score=driver+∑PC1 driver+∑PC1 suppressor. The FRGs score of each patient was calculated based on the formula and next normalized range from 0 to 1. These patients were classified into high or low score groups by best cutoff value. The Kaplan–Meier survival analysis was used to compare the different outcomes between high- and low score groups. To prove the robustness of the result, the FRGs signature was further validated in GSE19345 and GSE65904 datasets. Besides, to evaluate the prognostic value of FRGs score, multivariate cox regression for the overall survival (OS) time was performed on the traditional clinical factors and the FRGs score in multiple datasets. The hazard ratios (HR) and 95% confidence intervals (95% CI) of the prognostic factors were calculated.

### 2.6. Somatic Mutation Profile Analysis

The mutation profile of the TCGA-SKCM dataset was deposited in the form of Mutation Annotation Format at The Cancer Genome Atlas (TCGA) data portal (https://gdac.broadinstitute.org), which was analyzed and summarized by using the “Maftools” package. Firstly, the mutation landscape of low- and high-FRGs score subgroups was illustrated by oncoPrint plots. The top 10 most frequently mutated genes, as well as several well-known mutants in melanoma such as BRAF, NRAS, KRAS, HRAS, and NF1 between low- and high- FRGs score subgroups, were investigated. Besides, tumor mutational burden (TMB), which was defined as the number of mutations per megabase of the panel sequences examined, was calculated by Maftools. Next, the correlation between TMB and FRGs scores was also explored.

### 2.7. Immune Cells Infiltration and Immune Checkpoint Regulators Association

In order to investigate the association between FRGs score and immune status, the proportion of 22 kinds of immune cells in the tumor microenvironment was calculated via the CIBERSORT algorithm. Samples with CIBERSORT *P* < 0.05 were included in the correlation analysis between FRGs score and immune cells. In addition, to explore the potential biological phenotypes between high- and low-FRGs score groups, the expression data of immune checkpoint regulators was extracted and analyzed with FRGs score.

### 2.8. Gene Set Enrichment Analysis

To explore the signaling pathways enrichment for different FRGs score phenotypes in melanoma, Gene Set Enrichment Analysis (GSEA) was performed between low- and high-FRGs score groups. The cancer hallmark database (h.all.v7.0.symbols) and KEGG database (c2.cp.kegg.v7.0.symbols) were applied in GSEA to investigate the signaling pathways correlated with different subgroups of melanoma. The adjusted *P* < 0.05 were used to sort the significant pathways enriched in each phenotype.

### 2.9. Chemotherapy Drugs Prediction

To explore the likelihood of chemotherapeutic drugs, the CellMiner database (https://discover.nci.nih.gov/cellminer/) was performed to assess the correlation between FRGs score and drug response [16]. Firstly, the expression profiles of FRGs in NCI-60 cell lines and the drug activity were downloaded from the CellMiner database. Next, the FRGs score of each cell line was estimated and normalized accordingly. Finally, the Spearman test was applied to calculate correlation coefficients and *P* < 0.05 and |Correlation| > 0.25 were considered statistically significant.

### 2.10. Statistical Analysis

Statistical analyses in our study were conducted using R software version 3.6.0 with packages. The consensus clustering method was applied by the ConsensusClusterPlus package. Boruta algorithm was conducted by “Boruta” package, CIBERSORT algorithm was estimated by “CIBERSORT” package, GSEA was performed by using “clusterProfiler” package. The best cutoff values of each dataset were computed by using the “survminer” package. The Kaplan–Meier and Cox regression analyses were deployed by “survival” package. “prcomp” function in R was used to estimate PCA. The Spearman coefficient examined the correlation analyses. Wilcoxon tests were used to compare the difference in subgroups. The chi-square test analyzed the association between the FRGs score subgroups and somatic mutations. *P* < 0.05 or adjusted *P* < 0.05 suggests statistical significance in all tests.

## 3. Results

### 3.1. Differently Expressed FRGs

**Figure 1:**
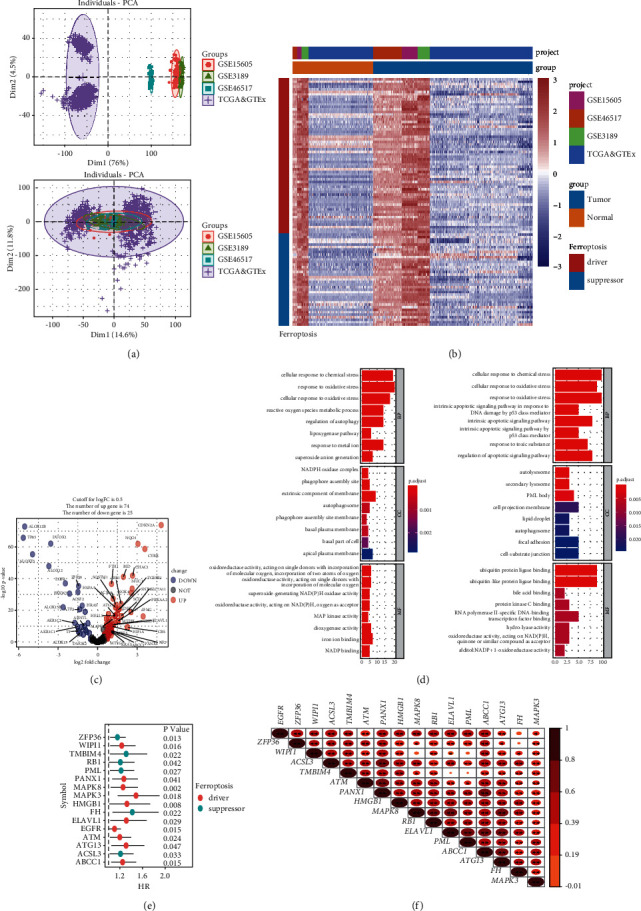
Selection of the prognostic value of differently expressed ferroptosis-related genes (FRGs). (a) The principal component distribution of datasets (TCGA-SKCM, GSE15605, GSE3189, and GSE46517) in before and after removal batch effect. (b) Heat map of differently expressed FRGs in the meta-cohort (TCGA-SKCM, GSE15605, GSE3189, and GSE46517). Rows represent FRGs and columns represent samples; red and blue indicate higher expression and lower expression. (c) Volcano plot of differently expressed FRGs in meta-cohort (FRGs with logFC ≥ 2 were labeled). (d) Gene Ontology (GO) enrichment analysis of the upregulated and downregulated FRGs. (e) Forest plots of 16 prognostic differently expressed FRGs. (f) Correlation analysis of 16 prognostic differently expressed FRGs. ^*∗*^*P* < 0.05; ^*∗∗*^*P* < 0.01.

A total of 870 samples were merged into a large cohort from the meta-cohort (TCGA-SKCM, GSE15605, GSE3189, and GSE46517), which consisted of 579 melanoma samples and 291 normal skin samples. The ComBat algorithm was performed to reduce the batch effect generated from the different platforms, and the PAC plot showed that the clusters based on the removal batch effect placed more together than before removal (Figure 1(a)). According to the selection standard, 99 differently expressed FRGs were screened out from the large cohort, where 74 FRGs were significantly upexpressed and 25 FRGs were significantly downexpressed in melanoma (Figure 1(c)). The heat map of differently expressed FRGs is illustrated in Figure 1(b). The significantly enriched signal pathways in the GO database are manifested in Figure 1(d).

### 3.2. Construction of the FRGs Classifier

**Figure 2:**
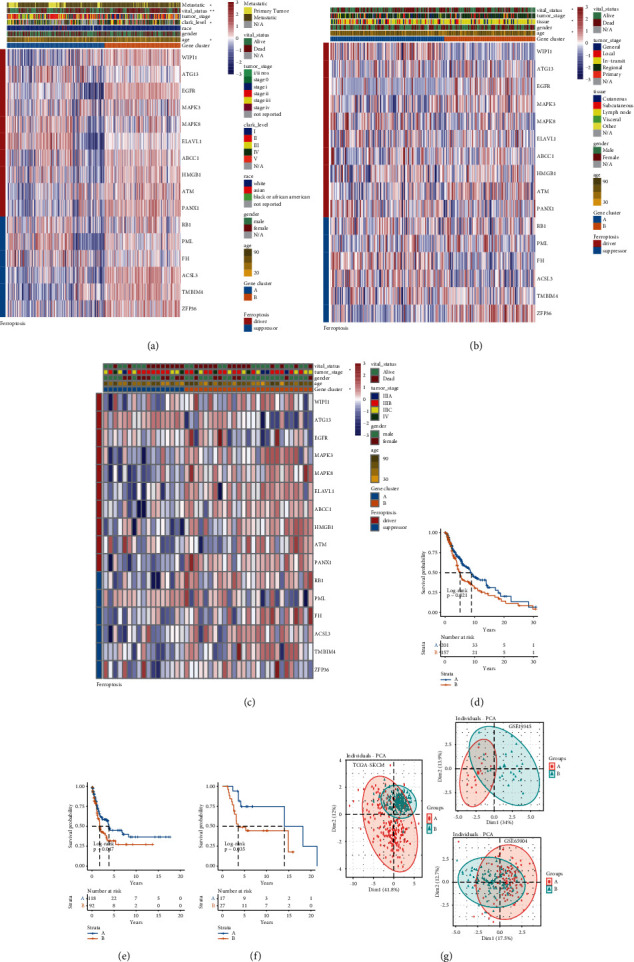
Consensus clustering analysis of prognostic ferroptosis-related genes (FRGs) in melanoma. (a) Unsupervised clustering of prognostic FRGs to classify patients into two groups in TCGA-SKCM. (b) Unsupervised clustering of prognostic FRGs in GSE19345. (c) Unsupervised clustering of prognostic FRGs in GSE65904. Heat maps manifested the correlation between the expression of FRGs and the clinical characteristics of the two melanoma clusters: cluster A (blue) and cluster B (orange). The color codes for different clinical parameters are as indicated. Red color indicates upregulation of FRGs, and blue color refers to downregulation of FRGs. (d) Kaplan–Meier (KM) analysis of cluster A and cluster B in TCGA-SKCM with log-rank *P*=0.02. (e) KM analysis of cluster A and cluster B in GSE19345 with log-rank *P*=0.035. (f) KM analysis of cluster A and cluster B in GSE65904 with log-rank *P*=0.035. (g) Principal component analysis (PCA) of prognostic FRGs expression profiles in TCGA-SKCM, GSE19345, and GSE65904 datasets, which illustrated distinct two clusters, cluster A (in red) and cluster B (in green).

Firstly, the prognostic value of these differently expressed FRGs was estimated by using univariate Cox regression analysis in TCGA-SKCM. The forest plot revealed that 16 FRGs that contained 9 driver and 7 suppressor genes were significantly associated with OS time (*P* < 0.05) (Figure 1(e)). The expression level of these FRGs in TCGA-SKCM was extracted for subsequent analysis, and the heat map of correlation demonstrated that these FRGs were strongly and positively associated with each other (Figure 1(f)). Next, we divided the melanoma patients into cluster A and cluster B based on the expression of corresponding FRGs (Figure 2(a)). The Kaplan–Meier curves suggested that the melanoma patients in cluster B have a poor survival outcome than the patients in cluster A with log-rank *P*=0.02 (Figure 2(d)). The stratified analysis for clinical variables indicated that vital status, metastatic status, clark level, and age in clusters A and B have significant differences. No correlations were observed in other variables such as gender, tumor stage, and race (Figure 2(a)). Moreover, patients in GSE65904 (Figure 2(b)) and GSE19345 (Figure 2(c)) datasets were classified accordingly and the Kaplan–Meier analyses indicated a similar result. Compared to patients in cluster A, cluster B had significantly shorter OS time with log-rank *P*=0.035 in GSE65904 (Figure 2(e)) and GSE19345 (Figure 2(f)). We further explored the association between two clusters and the clinical characteristics of melanoma patients in GSE65904 and GSE19345, respectively. We astonishingly found that age and vital status were also correlated with FRGs classifier. Lastly, PCA plots proven that the FRGs classifier can successfully divided melanomas into subtypes with different clinical outcomes (Figure 2(g)).

### 3.3. Construction of the FRGs Score

**Figure 3:**
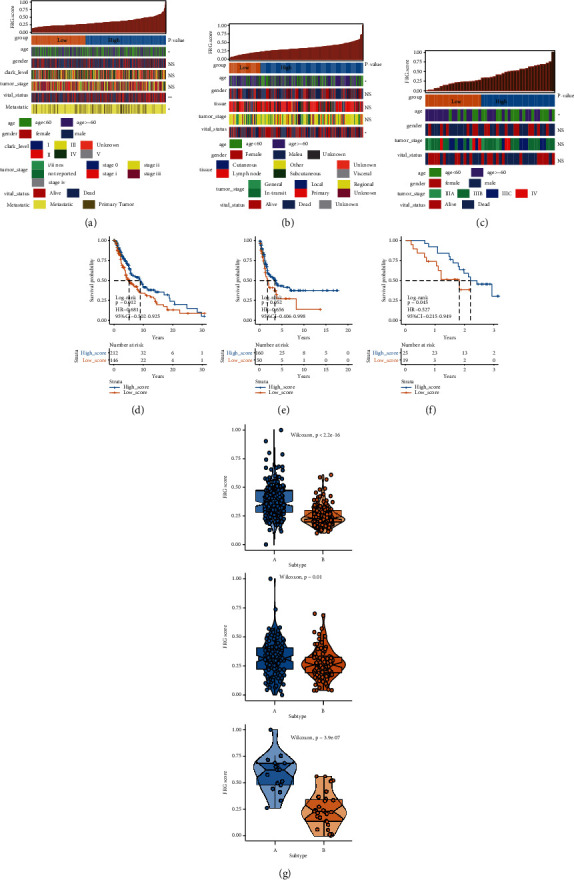
Clinical variables associated with the ferroptosis-related genes (FRGs) score in melanoma. (a) The association between FRGs score and clinical variables (age, gender, Clark level, tumor stage, vital status, and metastatic status) in TCGA-SKCM. (b) The association between FRGs score and clinical variables (age, gender, tissue, tumor stage, and vital status) in GSE65904. (c) The association between FRGs score and clinical variables (age, gender, tumor stage, and vital status) in GSE19345. ^*∗*^*P* < 0.05; ^*∗∗*^*P* < 0.01. (d) Kaplan–Meier (KM) analysis of the prognostic model for the 15-FRGs predictor in TCGA-SKCM. (e) KM analysis of the prognostic model for the 15-FRGs predictor in GSE65904. (f) KM analysis of the prognostic model for the 15-FRGs predictor in GSE19345. (g) The FRGs score distribution of subtype (cluster A and cluster B in TCGA-SKCM, GSE19345, and GSE65904 datasets.

To acquire the optimal gene indicator of FRGs in melanoma, we firstly used Boruta algorithm to screen FRGs based on the importance of genes. Nest, 15 FRGs (WIPI1, ATG13, EGFR, MAPK8, ELAVL1, ABCC1, HMGB1, ATM, PANX1, RB1, PML, FH, ACSL3, TMBIM4, and ZFP36) remained through this step and were subjected to FRGs score estimation. Based on the formula for FRGs score calculation, a FRGs score for each patient in TCGA-SKCM dataset will be generated. Then, by applying the best cutoff value, melanoma patients were divided into high score group (*n* = 212) and low score group (*n* = 146) in the TCGA-SKCM dataset. The distributions of the FRGs score, FRGs score subgroup, age subgroup, gender, Clark level, tumor stage, vital status, and metastatic status of patients in the TCGA-SKCM dataset are illustrated in Figure 3(a). The stratified analyses indicated that age, vital status, and metastatic status were intimately correlated with FRGs score. The Kaplan–Meier curves showed that patients in high score group have a longer survival time than low score with a log-rank test of *P*=0.012, 95% CI = 0.502–0.925 (Figure 3(d)). In addition, to prove the robustness of the result, validation analysis was performed in GSE65904 and GSE19345 datasets. The patients in GSE65904 and GSE19345 were classified into high score group or low score group according to TCGA-SKCM. The distributions of the FRGs score, FRGs score subgroup, age subgroup, gender, tumor stage, and vital status of patients in GSE65904 and GSE19345 were, respectively, manifested in Figures 3(b) and 3(c). Stratified analyses also suggested that age and vital status were closely associated with FRGs score. More interestingly, the Kaplan–Meier curves revealed that significant differences of survival time exist in the high score and low score groups regardless in GSE65904 (log-rank *P*=0.052, 95% CI = 0.406–0.998) (Figure 3(e)) or GSE19345 (log-rank *P*=0.045, 95% CI = 0.215–0.949) (Figure 3(f)). Besides, the violin plots manifested that the FRGs score in the cluster A subgroup was generally higher than that in the cluster B subgroup.

### 3.4. Independent Prognostic Value of the FRGs Score

**Figure 4:**
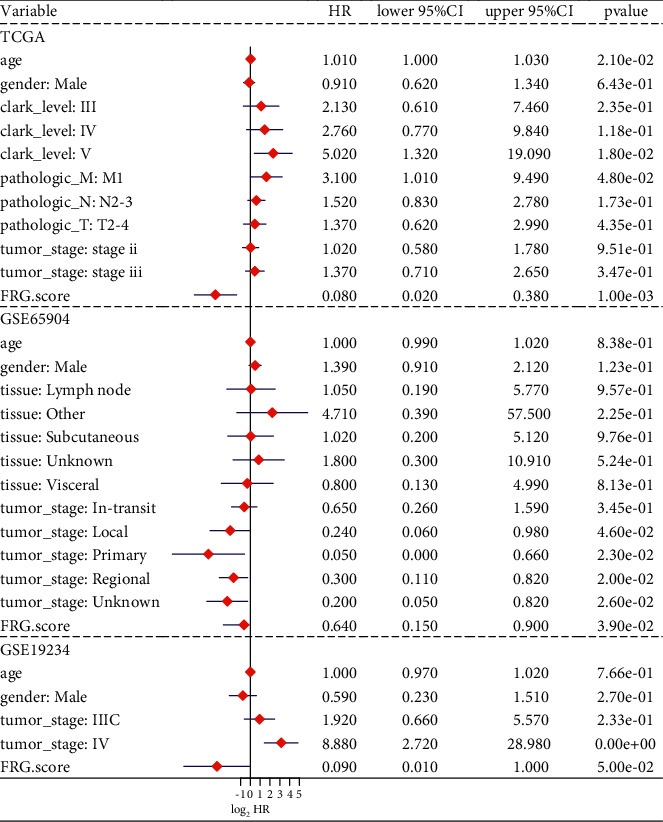
Multivariate Cox regression of ferroptosis-related genes (FRGs) score. Forest plot of multivariate Cox regression for FRGs score and clinical characteristics in TCGA-SKCM, GSE65904, and GSE19345 datasets.

To investigate the prognostic value for OS in multiple datasets, clinical variables and FRGs score were conducted by multivariate cox regression analyses (Figure 4). The forest plot suggested that only the FRGs score stably remained independent prediction for OS in TCGA-SKCM dataset (HR = 0.080, 95% CI = 0.02–0.038, *P*=0.001), GSE65904 dataset (HR = 0.640, 95%CI = 0.150–0.900, *P*=0.039), and GSE19345 dataset (HR = 0.090, 95%CI = 0.010–1.000, *P*=0.050).

### 3.5. Somatic Mutation in the FRGs Score Subgroup

**Figure 5:**
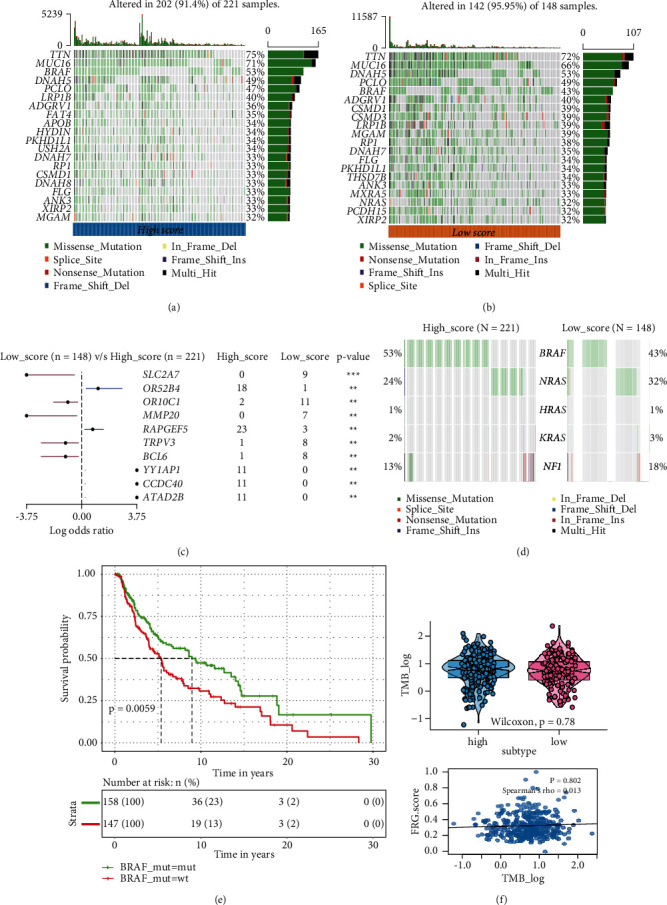
Mutational landscape between high and low ferroptosis-related genes (FRGs) score groups in TCGA-SKCM. (a) The oncoPrint of top 20 mutant genes in the high-FRGs score group. (b) The oncoPrint of top 20 mutant genes in low-FRGs score group. (c) Forest plots of somatic variants between high- and low-FRGs score groups (^*∗∗*^*P* < 0.01; ^*∗*^*P* < 0.05). (d) The oncoplots of mutants in BRAF, NRAS, KRAS, and NF1 between high- and low-FRGs score groups. (e) Kaplan–Meier (KM) analysis to compare overall survival (OS) between BRAF mutant and wild type. (f) The correlation and different analysis between FRGs score and tumor burden mutation (TMB).

To explore the potential association between somatic mutation and FRGs score, the Mutation Annotation Format in TCGA-SKCM was processed by the “maftools.” Firstly, patients in TCGA-SKCM were classified into low- and high-FRGs score groups. Next, the oncoPrint plots summaries of the top 20 gene mutation information of these two subgroups (Figures 5(a) and 5(b)). The top 3 genes with the highest mutation frequencies in the high-FRGs score group were TTN (75%), MUC16 (71%), and BRAF (53%), while those in the low-FRGs score group were TTN (72%), MUC16 (66%), and BRAF (43%).

The most prevalent significantly mutated genes in melanoma such as BRAF, NRAS, KRAS, HRAS, and NF1 were also explored; the results indicated that the high-FRGs score group have lower mutation frequencies in NRAS, KRAS, and NF1 compared to the low-FRGs score group (Figure 5(d)). Moreover, the forest plot revealed that OR52B4, RAPGEF5, YY1AP1, CCDC40, ATAD2B (highly mutated in the high score) and SLC2A7, OR10C1, MMP20, TRPV3, BCL6 (highly mutated in the low score) were significantly different between the low- and high-score groups (Figure 5(c)). Although spearman coefficient and subgroup analysis suggested that the TMBs were not significantly correlated with FRGs score (Figure 5(f)). Patients in the high score group had relatively high mutation frequencies of BRAF and indicated a good survival outcome in the Kaplan–Meier analysis between mutant and wild type of BRAF subgroups (Figure 5(e)).

### 3.6. Immune Cells Infiltration and Immune Checkpoint Regulators Association

**Figure 6:**
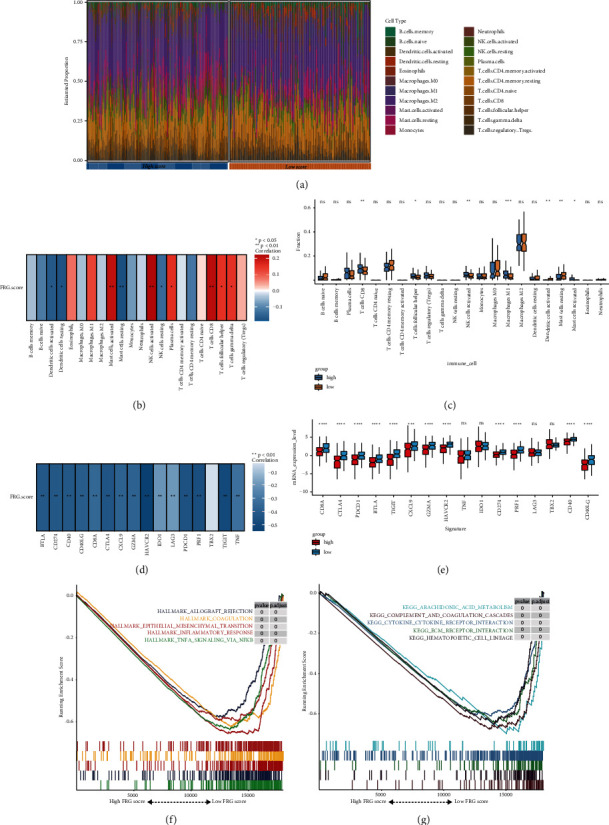
Tumor infiltrating landscape between high and low ferroptosis-related genes (FRGs) score groups in TCGA-SKCM. (a) The landscape of immune infiltration between high- and low-FRGs score groups. (b) Heat map of correlation analysis for the FRGs score and immune infiltrating cells. (c) The subgroup analysis of 22 immune infiltrating cells between high- and low-FRGs score groups. (d) The correlation analysis between FRGs score and the expression of immune checkpoints. (e) The subgroup analysis of immune checkpoints between high- and low-FRGs score groups; ^*∗*^*P* < 0.05, ^*∗∗*^*P* < 0.01, ^*∗∗∗*^*P* < 0.001,  and ^*∗∗∗∗*^*P* < 0.0001. (f) Cancer hallmark enrichment plots showed that allograft rejection, coagulation, epithelial-mesenchymal transition, inflammatory response, and TNFA signaling via NFKB were active in the low-FRGs score group. (g) KEGG enrichment plots manifested that arachidonic acid metabolism, complement and coagulation cascades, cytokine-cytokine receptor interaction, ECM receptor interaction, and hematopoietic cell lineage were active in the low-FRGs score group.

To explore the association between FRGs score and immune infiltration, the CIBERSORT algorithm was firstly used to comprehensively estimate the proportion of 22 immune cells in the immune microenvironment of melanoma. After the exclusion of low-quality samples, 207 melanoma patients were filtered out for further analysis. The distribution levels of 22 immune cells for the FRGs score subgroup in TCGA-SKCM are shown in Figure 6(a). Next, the correlation analyses indicated that FRGs score was positively correlated with Mast cells activated, NK cells activated, plasma cells, T cells CD8, T cells follicular helper, and T cells gamma delta, while being negatively associated with Dendritic cells activated and resting, Mast cells resting, and NK cells resting (Figure 6(b)). Subgroup analysis of 22 immune cells suggested that T cells CD8, T cells follicular helper, macrophages M1, NK cells activated, Mast cells activated and resting, and Dendritic cells were significantly different between high- and low-FRGs score groups (Figure 6(c)). Besides, to evaluate the association between FRGs score and immune checkpoint regulators, we selected the most prevalent immune checkpoint-relevant genes such as GZMA, CD40, CD40LG, LAG3, BTLA, PDCD1, IDO1, TIM3, CXCL9, CTLA4, HAVCR2, CD8A, TIGIT, CD274, PRF1, TBX2, and TNF for further analysis. We observed that almost all regulators were negatively correlated with FRGs score (Figure 6(d)). Except for regulators of TNF, IDO1, LAG3, and TBX2, all selected immune checkpoint-relevant genes were significantly overexpressed in the low-FRGs score group (Figure 6(e)).

### 3.7. GSEA

GSEA was performed to investigate the different signal pathways enriched in the high- and low-FRGs score groups. Based on the selection standard and the ranked pathways enriched in each phenotype, the top five pathways were illustrated in the GSEA plot. We observed that cancer hallmarks such as allograft rejection, coagulation, epithelial–mesenchymal transition, inflammatory response, and TNFA signaling via NFKB were all enriched in the low score group (Figure 6(f)). The KEGG results revealed that the low score group was significantly correlated with pathways such as arachidonic acid metabolism, complement and coagulation cascades, cytokine-cytokine receptor interaction, ECM receptor interaction, and hematopoietic cell lineage (Figure 6(g)).

### 3.8. Chemotherapy Drugs Prediction

**Figure 7:**
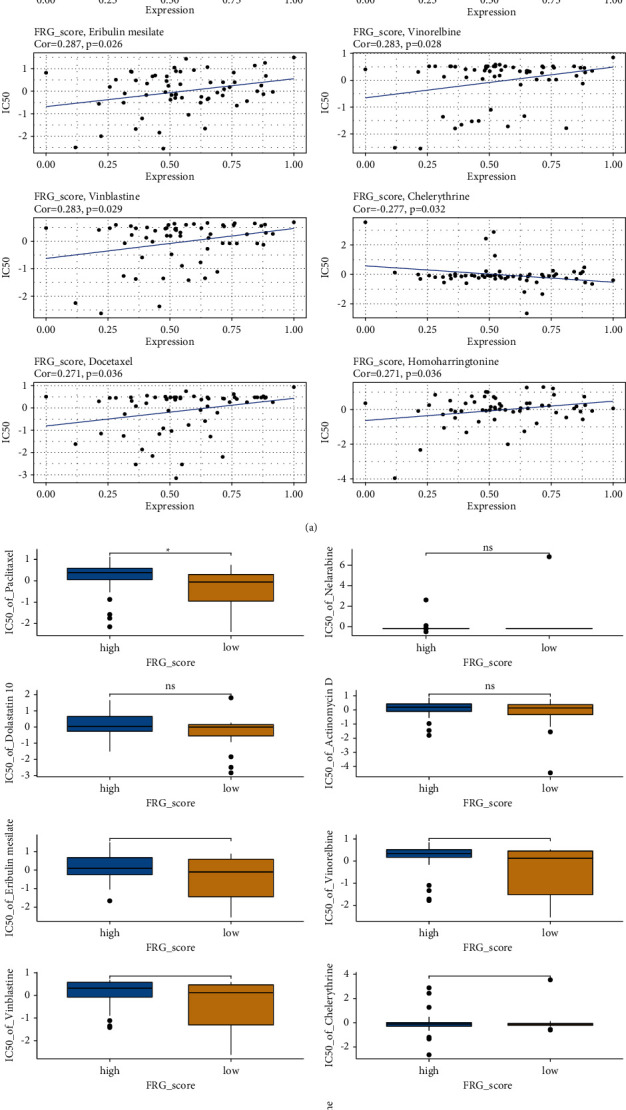
Relationships between ferroptosis-related genes (FRGs) score and chemotherapeutic response. (a) Correlation between the FRGs score and the IC50 value of drugs including paclitaxel, nelarabine, dolastatin 10, actinomycin D eribulin mesylate, vinorelbine, vinblastine, chelerythrine, docetaxel, and homoharringtonine. (b) The box plots of the estimated IC50 for paclitaxel, nelarabine, dolastatin 10, actinomycin D eribulin mesylate, vinorelbine, vinblastine, chelerythrine, docetaxel, and homoharringtonine between high- and low-FRGs score groups. ^*∗*^*P* < 0.05.

Currently, chemotherapy is effective for the treatment of melanoma. Herein, the CellMiner database was used to predict sensitivity to chemotherapy drugs. Eventually, we observed 10 drugs were significantly correlated with FRGs score, which included paclitaxel, nelarabine, dolastatin 10, actinomycin D, eribulin mesylate, vinorelbine, vinblastine, chelerythrine, docetaxel, and homoharringtonine (Figure 7(a)). Furthermore, the box plots manifested that the estimated IC50 of paclitaxel, vinorelbine, and vinblastine was significantly different between the high- and low-FRGs score groups (Figure 7(b)).

## 4. Discussion

Cutaneous melanoma is a heterogeneous disease with high metastases and death threat. The prognosis of melanoma is not only dependent on histological type but also relied on the molecular classification of cancer, which is critical to managing cancer with regard to diagnosis and therapeutic choice [18]. In addition, the traditional classification increasingly manifests ineffective and lack of benefits in clinical treatment. Hence, researchers are sparing no effort to investigate the novel molecular signature for better diagnosis and predicting prognosis. For example, BRAF mutations were generally observed in various types of cancer, including colon cancer, thyroid cancer, and melanoma [19–21]. The subtype cancer of the BRAF mutant usually benefits from inhibitors targeting this mutation. Recently, a growing amount of research suggested that ferroptosis working as a newly introduced cell death has shown a huge perspective of application in cancer treatment [22–24]. Therefore, we systematically analyzed the ferroptosis-related genes (FRGs) to identify two tumor subtypes with different clinical characteristics and establish a stable and precise signature for prognostic prediction in melanoma patients.

To our knowledge, this study is the first to explore the landscape of ferroptosis-related genes in cutaneous melanoma based on large cohort analysis. Through differently expressed gene analyses, we observed that the most of FRGs were up- or downexpressed in melanoma tissue. This activation of FRGs indicated that ferroptosis plays an important role in the progression of melanoma. Next, we performed GO enrichment and found that these differently expressed FRGs were positively correlated with oxidative stress pathway, lipoxygenase, apoptotic signaling, and iron ion binding, which reversely proven the reliability of our results.

Generally, a validity of classification is beneficial in predicting the clinical effects of genotyping with regard to treatment response [25]. Therefore, we identified two tumor subtypes (clusters A and B) of melanoma via the mRNA expression level of FRGs. Compare to the cluster A subtype, we observed that melanoma samples in cluster B have a shorter survival time and were closely associated with older age, higher Clark level, and high ratio of metastasis and death. It suggested that distinct differences of clinical and molecular characteristics existed in these two subtypes. Besides, the significant correlation between FRGs and OS in melanoma indicated that these prognostic FRGs were possibly used to build a model to predict the survival of patients. Afterward, we constructed and validated a robust 15-FRGs indicator to predict the prognosis of melanoma in multiple independent datasets. Our prognostic indicator can subsequently classify patients into subgroups with different survival events, somatic mutations, and immune infiltrations. Although no significant correlation was found between the TMB and FRGs score, high mutation of BRAF (53 vs. 43%) and low mutation of NRAS (24 vs. 32%), NF1 (13 vs. 18%) were observed in the high-FRGs score group. The mutant of BRAF in melanoma patients has predicted a good survival and the NRAS or NF1-mutant subtype of melanoma was associated with poor outcomes [26]. Recently, many studies have demonstrated that ferroptosis is intimately correlated with tumor immunity [27]. Based on GSEA results, we also discovered that many immune processes such as inflammatory response, TNFA signaling via NFKB, and cytokine receptor interaction were enriched in the FRGs score subgroup. It is logical to speculate that ferroptosis may have a close relationship with immunity in melanoma. Interestingly, we found that T cells (CD8+, follicular helper, gamma delta), activated Mast cells, NK cells, and Plasma cells were highly infiltrated in the high-FRGs score group, while almost all immune checkpoint-relevant genes involved in this study were highly expressed in low-FRGs score group. Previous research has proven that high infiltration of CD8+ T cells (adaptive immune response) and prominent infiltration of activated Mast cells, NK cells, and Plasma cells (native immune response) in malignant melanoma indicated a favorable prognosis [28]. Moreover, it is generally accepted that the high expression of immune checkpoints such as CTLA4, PD-1, BTLA, CD274, and LAG3 will benefit tumor cells to escape immune surveillance, avoid immune-mediated apoptosis, and finally lead to poor prognosis [29, 30]. Considered together, these results indicated that our indicator is reasonable and consistent with previous findings. More importantly, combined with multivariate cox regression analysis, we observed that the score of the 15-FRGs indicator can be considered as an independent prognostic model to afford a robustly accurate prediction of OS in melanoma patients.

Based on the annotation of the FerrDb website, our 15-FRGs indicator contained six suppressor genes (RB1, PML, FH, ACSL3, TMBIM4, ZFP36) that inhibit ferroptosis and nine driver genes (WIPI1, ATG13, EGFR, MAPK8, ELAVL1, ABCC1, HMGB1, ATM, PANX1) which promote ferroptosis. These genes are closely correlated with each other in melanoma. All of them have been proven to be associated with ferroptosis and even some of them have been widely researched in melanoma. For example, the retinoblastoma gene (RB1) regulated a series of malignant processes in melanoma cells, such as cell proliferation, differentiation, migration, and invasion [31]. Acyl-CoA synthetase long-chain family member 3 (ACSL3) is crucial to upregulate lipid caused by endoplasmic reticulum stress. Chen et al. reported that ACSL1 plays a potential oncogenic role in various tumors such as ovarian cancer, breast cancer, and melanoma [32]. Daniela et al. proved that WIPI1 is a melanoma-specific gene, which plays a key role in melanoma biology and could be regarded as a prognostic marker [33]. Moreover, it is well acknowledged that epidermal growth factor receptor (EGFR) is overexpressed in many solid tumors. Activation of EGFR will stimulate melanoma cells to progress or metastasize and be resistant to BRAF inhibitors [34, 35]. As for ATP binding cassette subfamily C member 1 (ABCC1), Chen et al. reviewed the previous literature and suggested that ABCC1 is a family member of ABC transporters and associated with the drug resistance of melanoma cells [36]. In brief, much previous research has manifested that these 15 genes may be closely correlated with ferroptosis in melanoma development and afforded a significant insight to construct a model based on FRGs.

Recently, ferroptosis is emerging as a promising approach for the treatment of cancer and especially tumor with conventional drug resistance. Therefore, exploring the potential chemotherapy drugs for inducing ferroptosis is a new therapeutic target for melanoma treatment. Through CellMiner database analysis, we discovered that paclitaxel, vinorelbine, and vinblastine were closely related to ferroptosis and have significantly different IC50 values between high- and low-FRGs score groups. Paclitaxel, as we know, is a chemotherapeutic agent widely used to treat solid tumors [37]. Even for the treatment of advanced melanoma, paclitaxel currently works as a second-line chemotherapeutic drug and provides the last choice for clinicians [38]. Vinorelbine is a semisynthetic vinca alkaloid, which kills tumor cells via mitotic apoptosis, autophagy, and inflammation [39]. Helen's team previously suggested that the combination between vinorelbine and IL-2 is considered as second-line therapy for metastatic melanoma [40]. Besides, vinblastine is a new form of vinca alkaloid. The combined chemotherapy containing cisplatin, vinblastine, and dacarbazine was universally used in many clinical trials and manifested encouraging results in advanced melanoma [41–43]. Apart from the three chemotherapy drugs, we also observed that seven drugs (nelarabine, dolastatin 10, actinomycin D, eribulin mesylate, chelerythrine, docetaxel, homoharringtonine) were closely associated with FRGs score. Hence, it is reasonable to assume that these chemotherapy drugs may be regarded as supplementary therapies or combined agents for the treatment of melanoma.

## 5. Conclusion

To sum up, our research provided a comprehensive analysis of ferroptosis for melanoma classification and constructed a robust 15-FRGs prognostic indicator which could be regarded as an independent prognostic model in clinical application. Furthermore, we also identified several potential chemotherapy drugs to induce ferroptosis and could supply additional effective treatments. The patients with the high-FRGs score suggest a good survival outcome and may acquire more chemotherapeutic benefits.

## Data Availability

The datasets generated for this study can be found in the GEO database (GSE15605, GSE3189 GSE46517, GSE19345, and GSE65904; https://www.ncbi.nlm.nih.gov/geo/), and UCSC Xena website (TCGA>Ex; https://gdc.xenahubs.net).
